# Military Urology

**Published:** 1918-09

**Authors:** 


### MILITARY UROLOGY

The American Red Cross in France, in publishing the Manual of Military Urology by Lt-Col. Hugh H. Young, Director of Urology of the American Expeditionary Forces, has not only shown its desire to serve the American Army but has presented to the world a really great contribution to the science and literature of war medicine.

Lt-Col. Young, and his collaborators, Lt-Col. E. L. Keyes, Captains M. L. Boyd, Edward L. Oliver, W. H. Mook and D. M. Davis and Lieutenants J. E. Moore and William Jack, have prepared in this book of 300 pages an authoritative discussion on the prevention and treatment of venereal diseases, including the surgery of the urinary and male genital organs, that will be of immense value to the surgeons of the American Expeditionary Forces in France. A section of the book is given over to dermatology, which is included in the Division of Urology of the Medical Department of the A. E. F.

The first two chapters, on the epidemiology and military control of venereal diseases, present very interesting data on their incidence in the British and French Armies and also among the troops of our enemies, the Germans and Austrians. They also give in detail the social and sanitary measures for preventing venereal diseases that have been adopted by the United States Army. The facts presented prove that our methods of handling this problem are not based upon moral and sentimental ideas, but that a practical working plan has been evolved, which will keep at a minimum the incidence of venèreal diseases among our troops, thus enormously increasing the strength and efficiency of our fighting forces.

In the chapter on the " Early Diagnosis of Syphilis " the authors claim that spirochetes can be demonstrated in the primary lesion in a very large proportion of all cases, unless the chancre has been previously treated with antiseptics; and state that " doubtful cases of the remaining 5 to 10 o/o must be regarded as possible syphilis ", and Wassermans made on them at the end of one and of two months.

The details of a standardized treatment for syphilis in the army are given and this is just as well adapted to treating syphilitics in civil life. Neosalvarsan (novarsenobenzol), manufactured by the French, has been adopted for army use because of the ease of administration, and the technic of administering it intravenously is described in detail. It is a modification of a French method by which soldiers can be treated without interfering with their work, except on the days of treatment. It therefore does away with the hospitalization of syphilitics, thus enormously decreasing the non-effective rate in the army.

The novarsenobenzol is dispensed in ampules of sufficient diameter so that a sterilized syringe can be introduced and the solution drawn out without having to be emptied in a glass vessel. It thus shortens the technic by one step, and lessens the chances for infection, since it is not necessary to sterilize a glass container for the solution.

The cyanide of mercury intravenously and Grey oil (40 o/o mercury) intra-muscularly are used in connection with the arsenic. The reactions from arsenic and mercury are described and the treatment for them outlined. The treatment and laboratory tests for the late syphilis are also discussed. Chancroid, other venereal ulcers, and inguinal adenitis are dealt with in a single chapter.

A routine examination of gonorrheal patients and a standard treatment for gonorrhea and its complications are described in detail. 5 cc. of 2 o/o protargol solution, injected into the anterior urethra 3 times a day, is used in acute gonorrhea until the purulent discharge is slight or non-purulent; and then a 5 o/o zinc sulphate solution or irrigation of permanganate of potassium, oxycyanide of mercury, or nitrate of silver 1 to 8000, increased in obstinate cases to a greater strength, even up to 1 to 2000, is substituted.

The section on dermatology is one of the most important in the book, since skin diseases are responsible for a large proportion of the non-effective rate in the army, and it is estimated that 90 o/o of them are preventable. Scabies, as was emphasized by speakers at the last meeting of the Research Society, is one of the commonest causes of temporary disability in all armies. Since the louse has been proved guilty of being the enemy that transmits typhus, relapsing and trench fevers, the de-lousing of soldiers, particularly after service in trenches that have been occupied by the Germans, has assumed considerable proportions as an army problem. The treatment, both curative and preventative, of scabies and pediculosis, as described in the Manual of Urology, has recently been carried out in several divisions of the A. E. F. with amazing results in reducing the prevalence of all skin diseases, thus considerably increasing the number of soldiers who are physically fit to serve at the front.

The appendix adds value to the book since it gives to the medical officers of the A. E. F. a permanent copy of many important letters and orders from the War Departement concerning venereal diseases. It also publishes the regulations concerning prostitution in France, and those regarding the sale or gift of alcoholic beverages to soldiers.

The Manual of Military Urology has been published by the American Red Cross in France for free distribution among the medical officers of the A. E. F. and of the armies of our Allies. The edition of 5000 copies has been nearly exhausted, but the type is being held to print another edition if there is a demand for it. Until the edition is exhausted, any medical officer of the A. E. F. or of the armies of our Allies may receive a copy on application to the American Red Cross Library, 12 place Vendôme, Paris.

